# PREDICTORS OF HIV STATUS DISCLOSURE AND CONDOM USE AMONG MOTHERS LIVING WITH HIV IN A TERTIARY HOSPITAL SOUTHEAST, NIGERIA.

**DOI:** 10.21010/Ajidv19i1.2

**Published:** 2024-10-25

**Authors:** ORJI Maria-Lauretta Chito, OKICHE Chikosolu Yvonne, AGBOEZE Joseph, JOE-AKUNNE Chinwe Ifeoma, OTUBO Sunday Austin, NWALI Matthew Igwe, ONYIRE Nnamdi Benson

**Affiliations:** 1 Consultant Paediatrics, Alex Ekwueme Federal University Ndufu-Alike, Ikwo Ebonyi state, Nigeria; 2 Consultant Obstetrician and Gynaecologist, Alex Ekwueme Federal University Teaching Hospital Abakaliki Ebonyi state, Nigeria

**Keywords:** Condom use, Mothers living with HIV, Prevalence, Serostatus, Status disclosure

## Abstract

**Background:**

Non-disclosure of HIV status and poor condom use, among mothers living with HIV may pose risks of HIV transmission to their serodiscordant partners and may influence the outcome of their infants. The study was aimed at assessing predictors of HIV status disclosure, and condom use, among mothers of infants exposed to HIV attending the ART clinic in Abakaliki, Southeast Nigeria.

**Materials and Methods:**

A hospital-based cross-sectional study that involved 246 mothers living with HIV. Information was obtained using an interviewer-administered structured questionnaire. Data was analysed using SPSS version 26 and p<0.05 was considered statistically significant.

**Results:**

A total of 190 (77.2%) of the 246 study participants disclosed their HIV status to their partners, but only 95 (38.6%) of them used condoms consistently and correctly during sexual intercourse. While 112 (45.5%) had HIV serodiscordant partners, 29 (11.8%) did not know the HIV status of their partners. Marital status of the mother (p=0.041), and HIV serostatus of the partner (p<0.001) having a child living with HIV (p=0.035) were predictors of HIV status disclosure to partners while HIV serostatus of the partner (p=0.041), the total number of children (p=0.031) and history of STDs (p=0.008) were predictors of condom use. Mothers with serodiscordant partners (AOR=2.21, 95%CI=0.94-11.32) and mothers with less than 4 children (AOR= 1.89, 95%CI= 0.52-8.33) were about twice more likely to use condoms compared to their colleagues.

**Conclusion:**

HIV serostatus of partners predicted status disclosure and condom use. Effective counseling is recommended to improve status disclosure and condom use especially among serodiscordant couples.

## Introduction

Globally, new HIV infections occur more commonly amongst serodiscordant partners in heterosexual relationships (Eyawo *et al.*, 2010). Prevalence of serodiscordance among heterosexual couples is high in sub-Saharan Africa and contributes to as high as 80% of new infections (Eyawo *et al.*, 2010). Transmission of HIV among heterosexual partners who do not know their HIV status is even higher; it is possible for HIV-infected women to erroneously assume that their sexual partners, whose HIV status are unknown to them, are also HIV-infected, as such, may not be cautious about condom use. There are evidences to show that HIV status disclosure to partners reduces the risk of HIV transmission among partners, and encourages consistent condom use. (Prestage *et al.*, 2001; Pinkerton and Galletly, 2007). Disclosing HIV status to an understanding partner enables the index partner to cope with the infection, commence ART early and adhere to it, hence it is vital to control of HIV/AIDS globally (Jasseron *et al.*, 2013). Despite these obvious benefits of HIV status disclosure, many HIV-infected women do not disclosure their HIV status to partners for reasons such as divorce/separation and fear of stigmatization. Some workers noted that HIV status disclosure was significantly higher among sero-concordant partners compared to sero-discordant partners (Stutterheim *et al.*, 2011; Ujah *et al.*, 2015; John and Chipwaza, 2022). This was corroborated by Mosisa *et al*, (2022) that reported prevalence of sero-status disclosure to be 73.77% and the determinants included knowing the partner’s status, and married, good level of education, long duration of antiretroviral (ART). This was similar to the findings of other authors like Olagbuji *et al*. (2011)

HIV status disclosure to partners promotes safe sex practices through consistent use of condoms (Pinkerton and Galletly, 2007). Consistent and correct use of condoms was noted to reduce the risk of HIV transmission during vaginal and anal intercourse and this is a key method used by discordant couples to reduce their risk (Prestage *et al.*, 2001; Pinkerton and Galletly, 2007) Previous authors, Adebayo *et al*. (2014) noted that marital status and secondary education were determinants of status disclosure and condom use respectively, but reported no relationship between status disclosure and consistent condom use. Epidemiological studies exist on serodiscordance and status disclosure, serodiscordance and condom use, or status disclosure and condom use. There is a dearth of information on the possible relationships that may exist between the three of them; status disclosure, serostatus, and condom use.

This study was therefore aimed at assessing the predictors of status disclosure and condom use in mothers of infants exposed to HIV. Information obtained from the study may guide in informed psychosocial support for mothers living with HIV to enable disclosure status and regularly use condoms.

## Materials and Methods

It was a hospital-based descriptive cross-sectional study, conducted in the Paediatric antiretroviral therapy (ART) clinic of Alex Ekwueme Federal Teaching Hospital Abakaliki (AEFUTHA), Ebonyi State. The clinic caters to most of all HIV-infected children and adolescents as well as HIV-exposed infants in Ebonyi State, Nigeria. The duration of the study was seven months (June to December 2022).

Sample size calculation: The sample size is calculated using the formula for descriptive study of a single population (Pourhoseingholi *et al.*, 2013). Using the prevalence rate of serodiscordant (80%) among HIV-infected mothers reported by Olagbuji *et al*. (2011) a power of 80%, a confidence interval of 95%, and a precision of 95%, a minimum sample size of approximately 246 was obtained

Subject selection: Mothers of infants exposed to HIV, who presented to the ART clinic with their infants were approached for the study and only those who gave informed consent were consecutively recruited into the study until minimum sample size was reached. An interviewer-administered questionnaire was used to obtain information on socio-demographics, literacy level, marital status, HIV status of their spouse, any HIV-infected child in the family, use of condoms, and history of sexually transmitted disease in the last year.

### Data analysis

The data was entered into SPSS version 26. Tables and charts were constructed as appropriate showing frequencies and percentages of variables. Categorical variables were analyzed using Pearson’s Chi-square and Fisher’s exact test for dichotomous variables. Multivariate Logistic regression model was used to determine the predictors of status disclosure and condom use among participants. The statistical significance was achieved at p= <0.05.

### Ethical Considerations

Ethical approval was obtained from the Health and Ethical Committee of Alex Ekwueme Federal Teaching Hospital before the commencement of the study. The HREC approval number is NHREC/16/05/22/132. The study was explained to participants and only those who gave written consent were included in the study. Confidentiality and privacy will be maintained throughout data collection and management.

## Results

The majority of the women recruited into the study were less than 35 years old (69.9%, 172/246) 193 of them were literate (78.5%), and the majority were married (87.4%, 215/246). A total of 120 (48.8%) of study participants were either public servants or privately employed. 154 (62.5%) had less than four children, 39 (15.9%) of them admitted to having had sexually transmitted disease in the last year and 35 (14.2%) of the study participants have at least one HIV-infected child. [Table 1]

**Table 1:** Socio-demographic characteristics of study participants

Variables	Frequency	Percentage (%)
Age (in years)		
<35	172	69.9
≥35	74	30.1
Mother’s literacy level		
Primary or less	53	21.5
Secondary or more	193	78.5
Occupation of mothers		
Private and public civil servant	120	48.8
Trader	63	25.6
Farmer	48	19.5
Housewife	15	6.1
Partner’s literacy level		
Primary or less	48	19.5
Secondary or more	198	80.5
Marital status		
Married	215	87.4
Divorced	7	2.8
Single	24	9.8
History of sexually transmitted disease		
Yes	39	15.9
No	207	84.1
Total number of children		
Less than 4	154	62.6
More than 4	92	37.4
Have HIV infected child or children		
Yes	35	14.2
No	211	85.8

**Figure 1:**
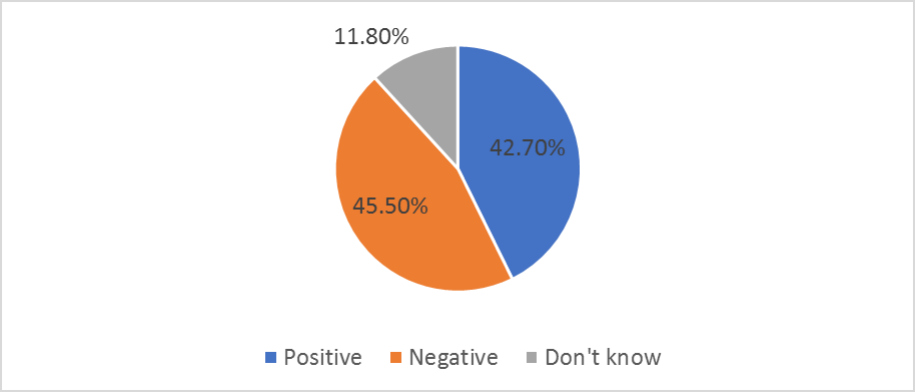
HIV serostatus of partners of study participants

A total of 105 (42.7%) out of the 246 women living with HIV were seroconcordant couples; while 112 (45.5%) reported having serodiscordant partners; HIV negative partners. A total of 29 (11.8%) were unaware of their partner’s HIV status as shown in Figure 1.

A total of 105 (42.7%) out of the 246 women living with HIV were seroconcordant couples; while 112 (45.5%) reported having serodiscordant partners; HIV negative partners. A total of 29 (11.8%) were unaware of their partner’s HIV status as shown in Figure 1.

HIV discordant partners were more among mothers living with HIV; less than 35 years of age (45.9%, 79/172); had education up to secondary school and more (47.7%, 92/193), and were divorced (57.1%, 5/7). The majority of the single parents (62.5%, 15/24) did not know the HIV status of their partners. Serodiscordant couples (62.9%, 22/35) had the highest frequency of having children living with HIV. There were significant relationships between the literacy level of the mother (y^2^ =6.12, p=0.047), marital status of the mother (y^2^ =41.62, p<0.001), having a child living with HIV (y^2^ =6.67, p=0.036) and HIV status of partner. [Table 2]

**Table 2:** Relationship between HIV Serostatus of partner and variables

Variables of Mothers Living with HIV	HIV Serostatus of partner			χ^2^	P value
	Positive (%)	Negative (%)	Unknown (%)		
Age (in years)					
<35	74 (43.0)	79 (45.9)	19 (11.1)	0.30	0.859
≥35	31 (41.9)	33 (44.6)	10 (13.5)		
Literacy level					
Primary or less	30 (56.6)	20 (37.7)	3 (5.7)	6.12*	0.047
Secondary or more	75 (38.8)	92 (47.7)	26 (13.5)		
Marital status					
Married	97 (45.1)	104 (48.4)	14 (6.5)	41.62*	<0.001
Divorced	3 (42.9)	4 (57.1)	0 (0.0)		
Single	5 (20.8)	4 (16.7)	15 (62.5)		
Any child living with HIV					
Yes	8 (22.9)	22 (62.9)	5 (14.2)		
No	97 (46.0)	90 (42.7)	24 (11.3)	6.67	0.036
Total number of children					
Less than 4	59 (38.3)	74 (48.1)	21 (13.6)	3.61	0.164
4 or more	46 (50.0)	38 (41.3)	8 (8.7)		

* Fisher’s exact test

**Figure 2:**
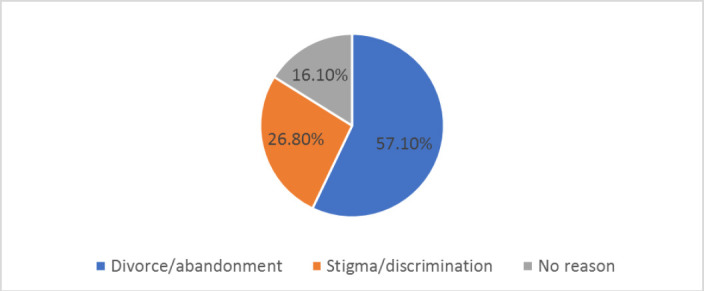
Reasons for HIV status non-disclosure to their partners

The majority (190/246, 77.2%) of the study participants have disclosed their HIV status to their spouses, while only 56 (22.8%) have yet to disclose their HIV status to their partners. Reasons for non-disclosure of serostatus include divorce/abandonment (57.1%), fear of stigma/discrimination (26.8%), and 16.1% giving no reason for non-disclosure. [Figure 2]

HIV status disclosure to spouse was noted to be highest among divorcees (85.7%, 6/7) and least among single mothers (29.2%, 7/24). While the majority (96.2%, 101/105) of seroconcordant mothers had disclosed their HIV status to their spouse, only 13.8% (4/29) of women whose HIV status of partners was unknown to them have disclosed their HIV status to such partners. The majority of the children living with HIV (94.3%, 33/35) were from mothers who had disclosed their HIV status to their spouses. The marital status of the mother (p=0.041), the HIV status of the partner (p<0.001), and having a child living with HIV (p=0.035) were predictors of HIV status disclosure to partners. [Table 3]

**Table 3:** Multivariate analysis of HIV Status disclosure and variables among mothers

Variables of mothers	HIV status disclosure		Adjusted OR	P value
Living with HIV	Yes (%)	No (%)	(95%CI)	
Age (In years)				
<35	136 (79.1)	36 (20.9)	1.09 (0.98-3.42)	0.296
≥35	54 (73.0)	20 (27.0)		
Literacy level				
Primary or less	40 (75.4)	13 (24.5)	0.12 (0.06-0.51)	0.730
Secondary or more	150 (77.7)	43 (22.3)		
Marital status				
Married	177 (82.3)	38 (17.7)	---	
Divorced	6 (85.7)	1 (14.3)	0.24 (0.13-0.47)	0.041
Single	7 (29.2)	17 (70.8)	0.20 (0.09-0.51)	0.229
HIV status of Partner				
Positive	101 (96.2)	4 (3.8)	---	
Negative	85 (75.9)	27 (24.1)	0.01 (0.01-0.03)	<0.001
Not known	4 (13.8)	25 (86.2)	0.08 (0.02-0.14)	<0.001
Total number of children				
Less than 4	117 (76.0)	37 (24.0)	0.82 (0.44-1.53)	0.327
4 and above	73 (79.3)	19 (20.7)		
Any child living with HIV				
Yes	33 (94.3)	2 (5.7)	0.35 (0.12-0.82)	0.035
No	167 (79.1)	44 (20.9)		

**Figure 3:**
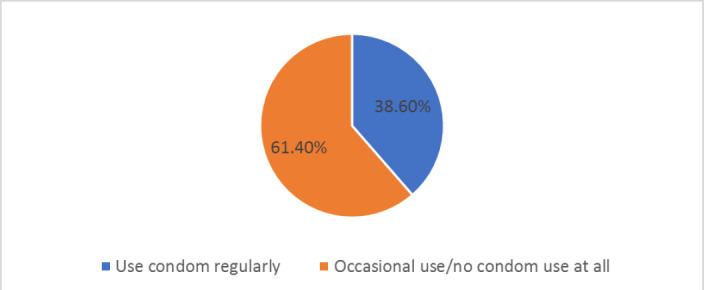
Condom use during sexual intercourse among study participants

The majority of the study participants did not use condoms or used condoms occasionally during sexual intercourse (61.4%, 151/246) compared to 95 (38.6%) who consistently used condoms during sexual intercourse [Figure 3]

Mothers with less than four children (44.0%, 68/154) had a higher prevalence of condom use compared to multiparous mothers. Also, condom use was more prevalent in literate mothers (39.9%, 77/193) and women who were unaware of their partner’s HIV status (55.2%, 16/29) compared to their counterparts. Thirty (76.9%) of the 39 women who reported sexually transmitted diseases in the last year did not use condoms at all or used them occasionally. The HIV serostatus of partners (p=0.041), total number of children (p=0.031), and history of STDs (p=0.008) were predictors of condom use in study participants. Mothers with less than four children (AOR=1.89, 95%CI= 0.52-8.33) and mothers with serodiscordant partners (AOR= 2.21, 95%CI= 0.94-11.32) were about twice more likely to use condoms compared to their counterparts. No significant relationship was observed between status disclosure and condom use among study participants. [Table 4]

**Table 4:** Multivariate analysis of condom use and variables among mothers living with HIV

Variables of the mothers living with HIV	Condom use among subjects		Adjusted OR (95%CI)	P value
	Yes (%)	No (%)		
Age (In years)				
<35	67 (39.0)	105 (61.0)	0.02 (0.01-0.62)	0.869
≥35	28 (37.8)	46 (62.1)		
Literacy level				
Primary or less	18 (34.0)	35 (66.0)		
Secondary or more	77 (39.9)	116 (60.1)	0.62 (0.33-1.97)	0.432
Marital status				
Married	85 (39.5)	130 (60.5)	----	
Divorced	2 (28.6)	5 (71.4)	0.22 (0.09-11.31)	0.137
Single	8(33.3)	16 (66.7)	1.18 (1.01-9.56)	0.168
HIV status of Partner				
Positive	30 (28.6)	75 (71.4)	----	
Negative	49 (43.8)	63 (56.2)	2.21 (0.94-11.32)	0.041
Not known	16 (55.2)	13 (44.8)	1.09 (0.67-2.89)	0.870
HIV status disclosure				
Yes	73 (38.4)	117 (61.6)		
No	22 (39.3)	34 (60.7)	0.59 (0.12-7.01)	0.734
Total number of children				
Less than 4	68 (44.2)	86 (55.8)	1.89 (0.52-8.33)	0.031
4 and above	27 (29.3)	65 (70.7)		
Have child living with HIV				
Yes	15 (42.9)	20 (57.1)	0.31 (0.14-0.53)	0.578
No	80 (37.9)	131 (62.1)		
History of STD				
Yes	9 (23.1)	30 (76.9)	1.08 (0.37-11.42)	0.008
No	86 (41.5)	121 (58.5)		

## Discussion

About half of the mothers living with HIV studied had serodiscordant partners. The prevalence found in index study was slightly lower than that obtained by Ujah *et al*. (2015) in Lagos (66.8%) and Eze *et al*. (2019) in Owerri (63.2%). However, a much lower prevalence was found in Plateau State (16.3%) (Magaji *et al.*,2018). The reason for the difference in prevalence rates may be as a result of the difference in location and sample size used. A significant number of divorcees living with HIV in the study had serodiscordant partners; this finding is similar to the observation made by Akpan *et al*. (2021) who noted that women with high discordant rates were more likely to be divorced/separated. The majority of the single mothers did not know the HIV status of their partners. This is not unexpected as many women who dwell in poverty-stricken regions of developing countries engage in sex for money, for survival and are not courageous enough to ask for the HIV status of their partner.

This study also found a significant relationship between literacy level, marital status, having children living with HIV, and serostatus of partner. Of note was that women with no formal education or whose highest level of education was primary school had the highest prevalence rate of HIV- positive partners. In a male-dominated society that permits polygamy and even the keeping of concubines, uneducated women are easy prey to such men due to ignorance and poverty. The serodiscordant couples were noted to have the highest number of HIV-positive children.

This buttresses the fact that in a serodiscordant relationship, the mother living with HIV may not be motivated to adhere to her ART medication, go for routine clinic visits, or even access maternal healthcare services during pregnancy and childbirth due to feeling of guilt, shame and fear of stigmatization (Amoran *et al.*, 2012)

The majority of the women studied had disclosed their status to their partners. This is very important as disclosure to partners encourages emotional and social support. This prevalence is similar to that by Ujah *et al*. (2015) in Lagos. The nondisclosure rate was also significant in the index study and the various reasons given by the women for nondisclosure included divorce or abandonment by partner, fear, and stigma. These reasons are not surprising as communal living is a major part of the culture in the study locality and HIV/AIDS is still a disease that is faced with stigma with people not wanting to associate with the carriers. Marital status, and having children living with HIV were found to be predictors of disclosure in the women we studied. Just like our study, Amoran (2012) in Ogun, and John and Chipwaza, (2022) in Tanzania had similar predictors for disclosure among other factors. HIV status disclosure was significantly higher in married couples compared to single partners. It is possible that these married women also had HIV-positive spouses as status disclosure was also found to be very high (96.2%) among seroconcordant couples. Studies (Ujah *et al.*, 2015; Mosisa *et al.*, 2022) have shown that seroconcordant couples are more open to themselves and cope better with the infection much more than serodiscordant couples. The reasons are obvious; they encourage one another to adhere to drugs, and clinic visits, and share emotional and social support. Divorcees had the highest prevalence rate of disclosure in the index study. One of the dreaded aftermaths of disclosure among married women is divorce or separation from their spouses. It is possible that the divorcee status was the consequence of HIV status disclosure to their partners. Mothers living with HIV who had disclosed their HIV status to their spouses were noted to have the highest prevalence of children living with HIV. It is plausible that this finding is a reflection of the majority of participants that had disclosed their HIV status to their spouses

Condom use among the women studied was low, only about 38.6% were using condoms consistently. This calls for concern for a study population that had about half of the participants as being serodiscordant and another 11.8% not knowing the HIV status of their partner. This result buttresses the need for more health education on preventive interventions especially during clinic visits. Other studies conducted in Nigeria showed low levels of consistent use of condoms, 38.4% in Portharcourt (Efobi *et al.*, 2021); 39.4% in Ogun (Ajayi *et al.*, 2022), and 29.4% in Abia (Nduka *et al.*, 2014). The prevalence of consistent condom use from our study was also similar to the prevalence found in studies done in other African countries such as Malawi (38.2%) (Haddad *et al.*, 2018) while high rate of consistent condom use 78.9% were reported in Ethiopia (Lotfi *et al.*, 2020). In the Ethiopia study, the male population contributed more to the higher rate of consistent condom use than their female counterparts. This probably indicates that there may be more gender and cultural factors that affect condom use. Mothers in a serodiscordant relationship were found to be twice as likely to use condoms compared to their counterparts. This is encouraging as condom use among serodiscordant couples has been reported to reduce HIV transmission in partners. This was alluded to by Eze *et al*. (2019) who noted that condom use was significantly associated with HIV serodiscordance.

Also from our study, we found that higher use of condoms was found among mothers with less than four children, women who did not know their partner’s status, and literate mothers compared to their counterparts. Other studies (Nduka *et al.*, 2014; Efobi *et al.*, 2021; Ajayi *et al.*, 2022), also alluded to the fact that literate mothers use condoms more consistently as they have better knowledge and understanding of its benefits. The majority of the mothers who reported sexually transmitted diseases either never used condoms or used them occasionally. This is not unexpected as correct and consistent use of condoms has been shown to prevent HIV including sexually transmitted diseases (Prestage *et al.*, 2001; Pinkerton and Galletly, 2007). The predictors of the use of condoms consistently were having less than four children and a positive history of previous sexually transmitted diseases (STD). These factors and predictors are similar to that found in studies done by Ajayi *et al*. (2022) and Nduka *et al*. (2014)

In conclusion, about half of the study participants had serodiscordant partners, while status disclosure was high with very low condom use. Serostatus of partners predicted HIV status disclosure and condom use among mothers living with HIV. There was however no relationship between status disclosure and condom use.

### Conflict of interest:

The Authors declare that there is no conflict of interest associated with this study.

**List of Abbreviations:**
AEFUTHA: Alex Ekwueme Federal University Teaching Hospital Abakaliki;
AIDS: Acquired immunodeficiency virus;
AOR: Adjusted odds ratio;
ART: Antiretroviral therapy;
HIV: Human immunodeficiency virus;
HREC: Health Research Ethical Committee;
SPSS: Social Package for Statistical Science;
STDs: Sexually transmitted diseases
